# Composite Complete-Blood-Count-Derived Inflammatory Indices for Discriminating Established Major Organ Involvement in Behçet’s Disease: A Cross-Sectional Nomogram Model

**DOI:** 10.3390/jcm15208067

**Published:** 2026-10-17

**Authors:** Suleyman Gul, Muammer Özdemir, Hilal Ecesoy

**Affiliations:** 1 Department of Physical Medicine and Rehabilitation, Karamanoğlu Mehmetbey University, 70100 Karaman, Türkiye; 2 Department of Medical Biochemistry, Karamanoğlu Mehmetbey University, 70100 Karaman, Türkiye; 3 Department of Rheumatology, Karamanoğlu Mehmetbey University, 70100 Karaman, Türkiye

**Keywords:** Behçet’s disease, major organ involvement, systemic inflammatory response index, nomogram, biomarkers, systemic vasculitis

## Abstract

**Introduction**: Differentiating major organ involvement (MOI) from isolated mucocutaneous manifestations in Behçet’s disease (BD) remains a substantial clinical challenge, particularly in stable outpatients outside acute flares. This study aimed to evaluate the cross-sectional discriminatory utility of complete blood count (CBC)-derived composite inflammatory indices—specifically the Systemic Inflammatory Response Index (SIRI), Systemic Immuno-Inflammatory Index (SII), and Aggregate Index of Systemic Inflammation (AISI)—and to construct an internally validated diagnostic nomogram. **Methods**: A total of 178 BD patients (88 with established MOI and 90 with isolated mucocutaneous involvement) classified according to the 2014 International Criteria for Behçet’s Disease (ICBD) were included in this cross-sectional study. Concurrently measured hematological parameters, acute-phase reactants (Erythrocyte Sedimentation Rate [ESR] and C-Reactive Protein [CRP]), and ongoing therapies were analyzed. Discriminative performance was evaluated using receiver operating characteristic (ROC) curves. Independent indicators of MOI were identified via multivariable Firth’s penalized logistic regression, and a nomogram was generated with 1000-sample bootstrap internal validation. **Results**: In the MOI group, SIRI, SII, and AISI levels were statistically significantly higher than in the mucocutaneous group (*p* < 0.001); in contrast, no statistically significant difference was observed in ESR and CRP levels (*p* > 0.05). SIRI provided the highest numerical point estimate for AUC (0.919, 95% CI: 0.880–0.956); however, formal matched ROC comparisons showed that SIRI’s discriminatory performance was statistically comparable to that of AISI (AUC: 0.916; *p* = 0.800) and SII (AUC: 0.889; *p* = 0.211). In a multivariable analysis adjusted for age, sex, and duration of illness, SIRI remained strongly and independently associated with MOI (adjusted OR: 90.19, 95% CI: 22.20–366.44, *p* < 0.001). The SIRI-based diagnostic nomogram demonstrated robust calibration and discriminatory power (optimism-adjusted AUC = 0.912, Brier score = 0.115). **Conclusions**: Conventional acute-phase reactants fail to reflect established MOI in BD patients receiving maintenance therapies outside acute flares. In contrast, composite CBC-derived indices—SIRI, SII and AISI—exhibit strong discriminative accuracy. The internally validated nomogram provides an accessible clinical stratification tool. However, the present cross-sectional data do not establish prediction of future MOI, prognosis, or subclinical vasculitis.

## 1. Introduction

Behçet’s disease (BD) is a chronic systemic vasculitis characterized by recurrent oral ulcers, genital ulcers, and uveitis and can affect multiple organ systems. Although the pathogenesis of the disease is not fully understood, it is more frequently observed in families with a history of Behçet’s and in individuals carrying the HLA-B51 allele, a genetic marker associated with it [1]. The pathophysiology of BD involves a thrombo-inflammatory process characterized by endothelial dysfunction, neutrophil hyperactivity, increased chemotaxis, and increased pro-inflammatory cytokines. In autoinflammation, the overactivation of innate immune cells (neutrophils, monocytes) and, in autoimmunity, the overactivation of the acquired immune response (especially Th17 cells and cytokine release) play a role in the disease [2].

The clinical presentation of BD is highly heterogeneous, ranging from recurrent mucocutaneous lesions—such as oral and genital ulcers—to severe major organ involvement (MOI), including vascular, ocular, neurological, and gastrointestinal complications that carry a high risk of morbidity and mortality [3,4,5,6,7,8,9,10]. In routine clinical follow-up, traditional acute phase reactants such as Erythrocyte Sedimentation Rate (ESR) and C-Reactive Protein (CRP) are frequently utilized; however, they cannot reliably detect subclinical inflammation or differentiate established major organ involvement [11,12].

This diagnostic challenge has driven the search for novel biomarkers. New-generation complete blood count (CBC)-derived cellular inflammation indices are inexpensive, widely accessible, and potentially useful tools in predicting systemic inflammatory load. However, the actual knowledge gap in the literature persists: while these parameters show promise, their incremental cross-sectional discriminatory value for established MOI and their transportability across different patient cohorts and clinical settings remain uncertain.

In the follow-up of BD, traditional acute phase reactants such as Erythrocyte Sedimentation Rate (ESR) and C-Reactive Protein (CRP) are used in clinical practice despite their low specificity [12]. However, these biomarkers cannot detect subclinical inflammation or differentiate major organ involvement. This situation leads to the search for new biomarkers in making early and aggressive treatment decisions in the management of the MOI phenotype. New-generation cellular inflammation indices derived from routine complete blood count parameters are attracting attention as low-cost, rapid and reliable alternatives in predicting systemic inflammatory load and disease activity in various rheumatological and autoimmune diseases. Previous studies have indicated that neutrophil–lymphocyte ratio (NLR), neutrophil–monocyte–lymphocyte ratio (NMLR) and platelet–lymphocyte ratio (PLR) can be determinants of vascular activity [13,14].

Neutrophils and platelets included in the inflammatory indices are conventionally proposed as surrogate markers for vascular stress and inflammatory activity; monocytes are linked to chronic inflammation; and the decrease in lymphocytes may reflect the body’s suppressed adaptive immune response to immune stress. There are a limited number of studies in the literature on the diagnostic value of these indices and ratios in Behçet’s disease, and especially their success in differentiating vascular complications [15,16,17,18]. This study aims to develop a nomogram-based risk stratification model with an internally validated nomogram using indicators derived from routine blood count parameters, thereby contributing to the cross-sectional identification of the defined MOI phenotype and the planning of effective treatment interventions.

## 2. Materials and Methods

### 2.1. Study Design and Participants

This study was designed as a cross-sectional study evaluating the clinical and laboratory data of patients diagnosed with Behçet’s disease (BD) according to the International Criteria for Behçet’s Disease (ICBD) 2014. The study included patients who presented to the Physical Medicine and Rehabilitation and Rheumatology outpatient clinics of Karaman Training and Research Hospital. Patients’ age, gender, disease duration, and medical treatments (colchicine, DMARDs [disease-modifying antirheumatic drugs], and biological agents) were recorded at the time of clinical evaluation.

**Figure 1:**
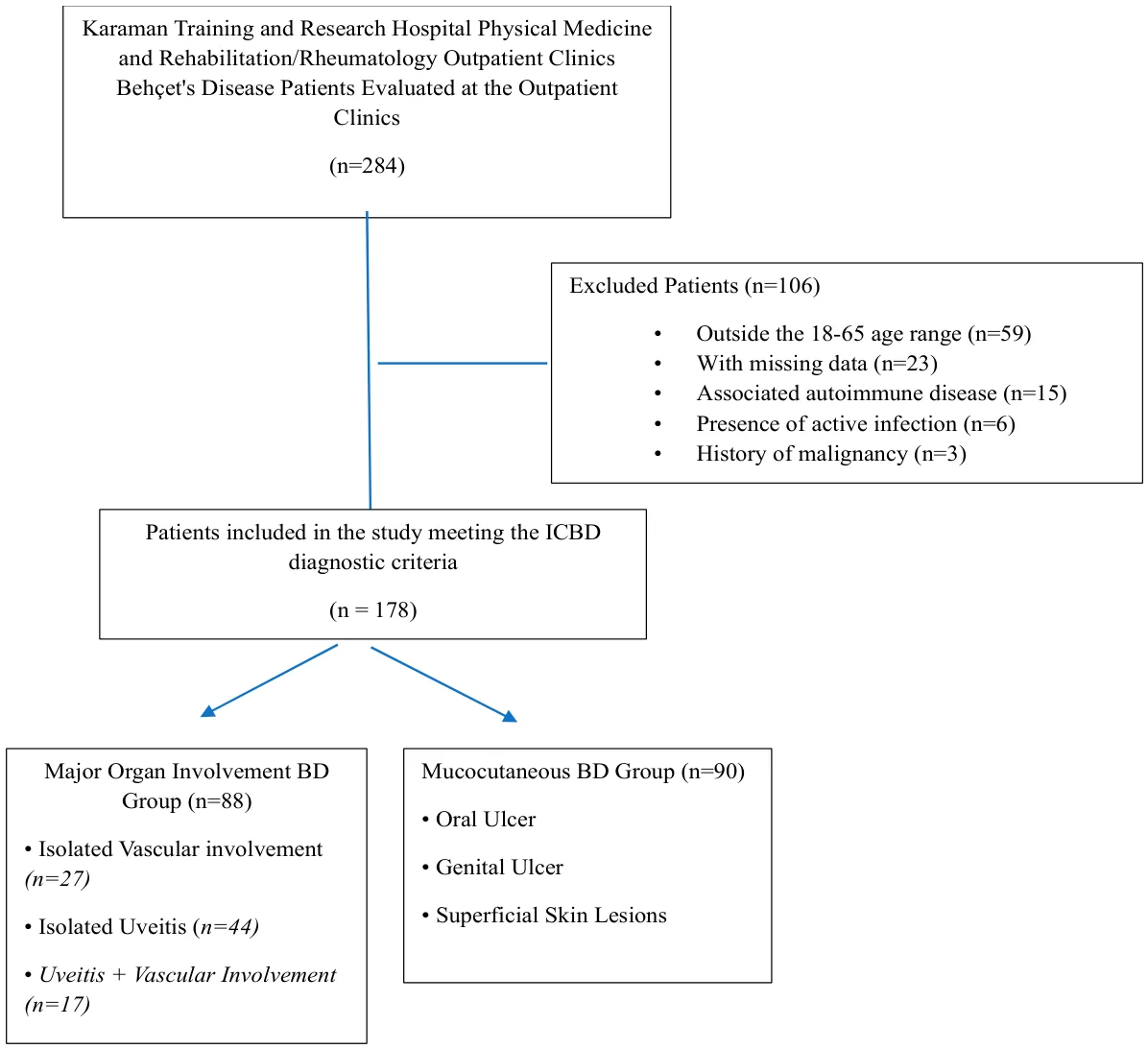
Patient recruitment flowchart showing the inclusion/exclusion criteria applied to the study population and the stratification of patients according to their clinical phenotypes based on major organ involvement. (BD: Behçet’s Disease, ICBD: International Criteria for Behçet’s Disease, n: Number of patients).

Patients with concomitant inflammatory diseases, infections, or malignancies, and those without available laboratory data, were excluded from the study. Importantly, to prevent profound pharmacological confounding on complete blood count parameters, patients receiving systemic glucocorticoid therapy at the time of evaluation and blood sampling were systematically excluded. A total of 284 patients were initially evaluated for eligibility. Based on the exclusion criteria, 106 patients were removed from the study. The study group was divided into two groups: MOI phenotype and mucocutaneous BD. The MOI phenotype BD group consisted of patients with vascular and ocular involvement. To ensure uniform classification, organ involvement was verified through formal diagnostic modalities: ocular involvement (recurrent anterior/posterior uveitis, retinal vasculitis, or panuveitis) was diagnosed and confirmed by a certified ophthalmologist via slit-lamp examination and fundoscopy; vascular disease (deep vein thrombosis, superficial thrombophlebitis, or arterial aneurysms/occlusions) was confirmed by Doppler ultrasonography or computed tomography angiography. Blood samples were collected during routine outpatient follow-up visits while patients were receiving maintenance immunomodulatory therapy, excluding periods of acute systemic flares. Consequently, the MOI in our cohort reflects established, clinically silent, or low-grade chronic disease phenotypes rather than an acute and debilitating presentation. The mucocutaneous BD group consisted of patients with oral ulcers, genital ulcers, and superficial skin lesions. Because current clinical guidelines do not recommend routine advanced systemic screening in asymptomatic patients, the absence of MOI in this group was determined based on comprehensive clinical history, physical examination, and the absence of organ-specific symptoms or clinical signs, rather than through universal standardized ophthalmological, vascular, neurological, or endoscopic evaluations (Figure 1).

### 2.2. Laboratory Measurements

Blood samples were drawn concurrently during routine outpatient follow-up visits, outside of acute flare-ups, while patients were on their ongoing maintenance treatments. Routine biochemical and hematological parameters recorded included complete blood count (neutrophil, lymphocyte, monocyte, and platelet counts), ESR, and CRP levels. Inflammation markers used in the study were calculated using complete blood counts according to the following formulas:

- Systemic Immuno-Inflammation Index (SII): Platelets × Neutrophils/Lymphocytes;
- Systemic Inflammation Response Index (SIRI): Neutrophils × Monocytes/Lymphocytes;
- Systemic Inflammation Aggregate Index (AISI): Neutrophils × Platelets × Monocytes/Lymphocytes;
- Neutrophil–lymphocyte ratio (NLR): Neutrophils/Lymphocytes;
- Platelet–lymphocyte ratio (PLR): Platelets/Lymphocytes;
- Neutrophil–monocyte–lymphocyte ratio (NMLR): (Neutrophils + Monocytes)/Lymphocytes.

### 2.3. Statistical Analysis

Statistical analyses were performed using Python (version 3.10, Python Software Foundation, Wilmington, DE, USA) and SPSS (version 22.0, IBM Corp., Armonk, NY, USA). The normality of continuous variables was assessed using the Shapiro–Wilk test. Since the variables did not exhibit a normal distribution, the median and interquartile range [IQR] are expressed. Categorical variables were presented as frequencies and percentages. Group comparisons for continuous variables were conducted using the Mann–Whitney U test for primary two-group analyses (e.g., overall MOI versus mucocutaneous phenotype) and the Kruskal–Wallis test for multi-group comparisons involving more than two categories (e.g., the four-group discrete clinical phenotype analysis). The Chi-square test was used for categorical variables. The Spearman rank correlation coefficient was used to evaluate the relationships between indices.

**Figure 2:**
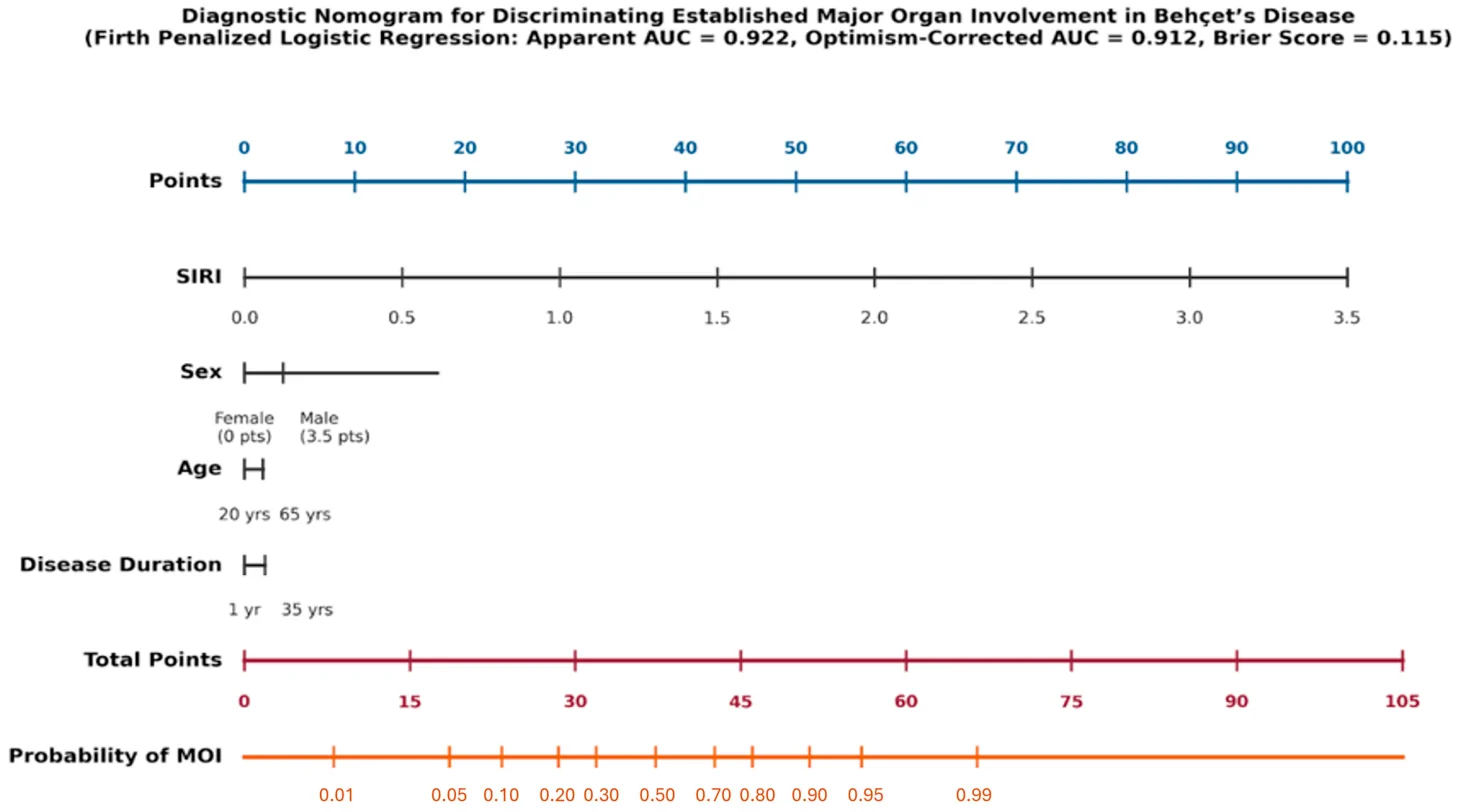
Diagnostic nomogram for discriminating established major organ involvement in Behçet’s disease.

Receiver Operating Characteristic (ROC) curve analysis was used to determine diagnostic performance and calculate the Area Under the Curve (AUC). Optimal cut-off values were determined using the Youden Index. Multivariable Firth’s penalized logistic regression was conducted to determine the independent association of SIRI with established MOI while adjusting for clinical covariates, including age, sex, and disease duration. To avoid potential post-outcome bias and collider bias, treatment variables were deliberately not entered into the core multivariable nomogram model. Because advanced therapies (such as biological agents and DMARDs) are prescribed directly consequent to the establishment of major organ manifestations and inherently alter leukocyte counts, attempting simple statistical adjustment for these treatments would severely distort the model’s validity. The nomogram was constructed based on this multivariable model (Figure 2). Internal validation was performed with 1000 bootstrap resamples to assess overfitting and calculate optimism-corrected performance.

## 3. Results

The study included 178 patients. Eighty-eight of these patients were grouped as having an MOI phenotype with vascular and ocular involvement. The mucocutaneous BD group consisted of 90 patients with oral ulcers, genital ulcers, and superficial skin lesions but without major organ involvement. There was no statistically significant age difference between the mucocutaneous BD (median: 42.50) and MOI phenotype BD (median: 44.00) groups (*p* = 0.196). This indicates that the differences in next-generation indices are not due to the age variable. In the MOI phenotype BD group, disease duration (median: 15 years) was significantly longer than in the mucocutaneous BD group (median: 12 years) (*p* = 0.018). The male gender ratio was statistically higher in the MOI group (*p* < 0.001).

The rate of colchicine use in mucocutaneous BD was 96.7%, while this rate decreased to 69.7% in the MOI phenotype BD group (*p* < 0.001). Biological agent use was 30.3% in the MOI phenotype BD group (0% in the mucocutaneous group), and DMARD use was 60.7% in MOI phenotype BD (25.6% in the mucocutaneous group) (*p* < 0.001). As per the study design, none of the 178 patients included in the final analysis were receiving systemic glucocorticoid therapy at the time of blood sampling.

ESR (*p* = 0.388) and CRP (*p* = 0.533), the most frequently measured acute-phase reactants in clinical practice, did not show a statistically significant difference across the clinical phenotypes.

All cellular indices derived from the complete blood count were found to be statistically significantly higher in the MOI phenotype BD group (*p* < 0.001). To comprehensively address the phenotypic heterogeneity of the disease, the MOI group was further stratified into isolated uveitis (*n* = 44), isolated vasculitis (*n* = 27), and combined uveitis and vasculitis (*n* = 17) phenotypes (Table 1).

Spearman correlation analysis revealed that next-generation cellular indices derived from routine complete blood count parameters exhibited strong and statistically significant positive correlations among themselves. Near-perfect correlations were found between SIRI and AISI (r = 0.91) and SII and AISI (r = 0.87), while complete agreement was observed between NLR and NMLR (r = 1.00). In contrast, the correlations between cellular indices such as SIRI, SII, and AISI and traditional acute phase reactants CRP (r = 0.11, 0.16, 0.17, respectively) and ESR (r = 0.05, 0.27, 0.19, respectively) were found to be clinically insignificant and extremely weak.

**Figure 3:**
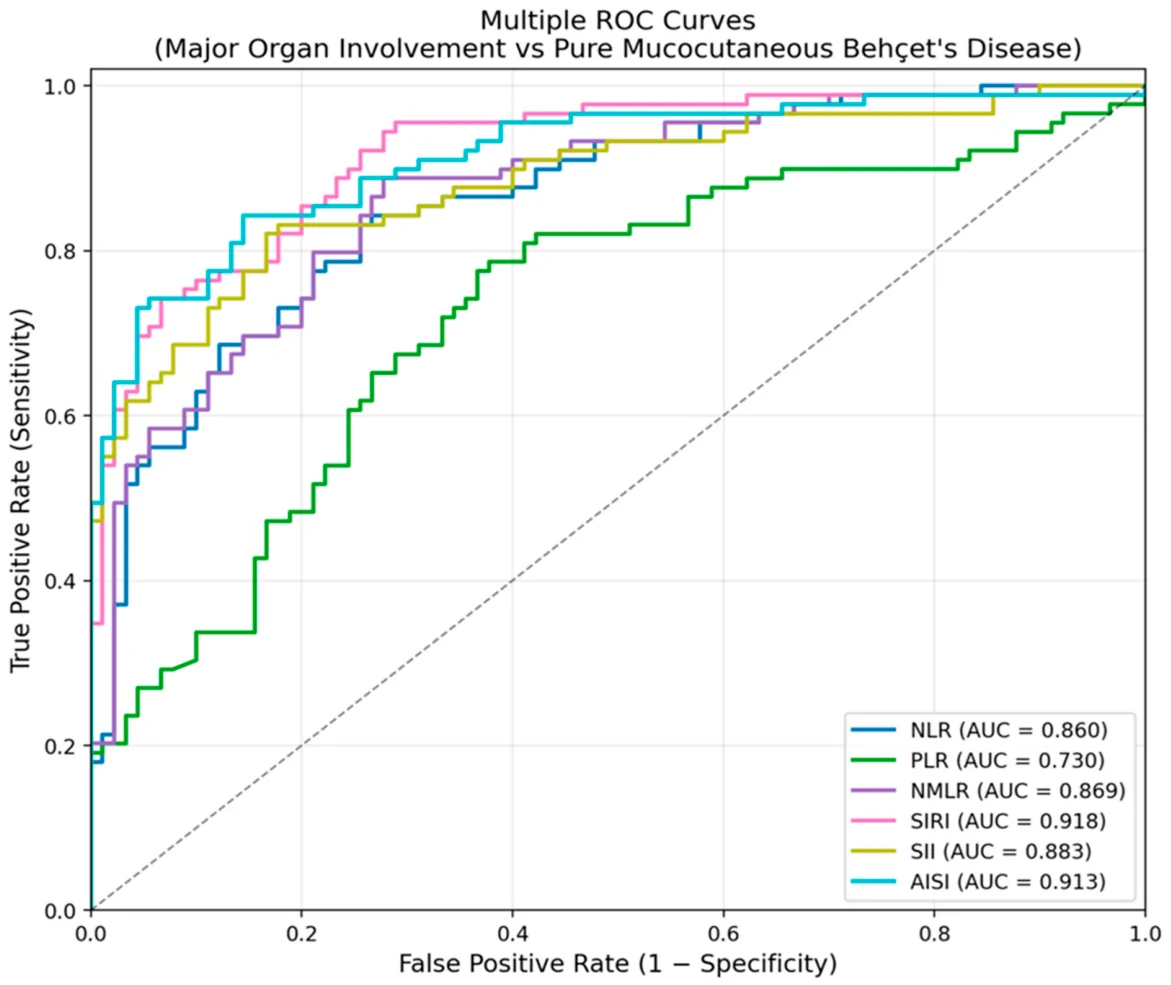
Evaluation of the diagnostic accuracy of the examined next-generation cellular indices in differentiating mucocutaneous and MOI BD phenotypes using ROC curve analysis. The gray dashed diagonal line represents the reference line of no discrimination (chance level, AUC = 0.50). Abbreviations: AUC: Area Under the Curve; SIRI: Systemic Inflammation Response Index; AISI: Systemic Inflammation Aggregate Index; SII: Systemic Immuno-Inflammation Index; MOI: Major Organ Involvement; NMLR: Neutrophil-Monocyte/Lymphocyte Ratio; NLR: Neutrophil/Lymphocyte Ratio; PLR: Platelet/Lymphocyte Ratio.

Our proposal should be as follows. “The discriminatory performance of the examined clinical and laboratory parameters in distinguishing between the mucocutaneous and MOI phenotypes in Behçet’s disease was evaluated using ROC curve analysis. he Area Under the Curve (AUC) values and optimal cut-off points calculated according to the Youden J statistic, along with sensitivity and specificity ratios for all parameters, are presented in Table 2. According to the analysis results, composite cellular indices (SIRI, AISI, SII) demonstrated substantially higher discriminative accuracy compared with conventional acute-phase reactants (*p* < 0.001) and individual hematological counts. Organ-specific subgroup ROC analyses confirmed that SIRI maintained excellent cross-sectional discriminative accuracy when differentiating purely mucocutaneous disease from isolated uveitis (AUC: 0.935) and from isolated vascular involvement (AUC: 0.924) (Table 2). This confirms that the robust global discriminative performance was consistent across discrete subphenotypes and not solely driven by a single dominant condition. In contrast, traditional markers commonly used in clinical practice to monitor disease activity and inflammation, such as CRP (AUC = 0.562) and ESR (AUC = 0.558), remained very close to the reference line (50%) with no discrimination in the ROC curve. Figure 3 shows the evaluation of the diagnostic accuracy of next-generation cellular indices in distinguishing between mucocutaneous and MOI phenotype BD phenotypes using ROC curve analysis.

To identify independent factors associated with the presence of the MOI phenotype, a fully adjusted Firth’s penalized multivariable logistic regression model was applied. The model included clinically relevant covariates: age, sex, disease duration, and concurrent treatments (colchicine, DMARDs, and biological agents). In multivariable Firth’s penalized logistic regression analysis, SIRI remained strongly and independently associated with the presence of established MOI after adjusting for age, sex, and disease duration (adjusted OR: 90.19, 95% CI: 22.20–366.44, *p* < 0.001) (Table 3). However, the very large adjusted odds ratio for SIRI should be interpreted cautiously. Because SIRI is a continuous variable with a relatively narrow biological range, a one-unit increase represents a massive shift across its distribution, mathematically inflating the odds ratio. This scale dependency, combined with the wide confidence interval, indicates that while the association is highly significant, the absolute magnitude of the point estimate is scale-specific. Based on this model, a diagnostic nomogram was constructed (Figure 3). The apparent AUC of the multivariable model was 0.922 (95% CI: 0.881–0.963). Internal validation using 1000 bootstrap resamplings demonstrated minimal optimism (0.0098), yielding a robust optimism-corrected AUC of 0.912 and a Brier score of 0.115. SIRI’s discriminatory consistency has been validated through pre-defined subgroup analyses demonstrating sustained diagnostic efficacy across all age and disease duration categories (Table 4).

To visually and practically estimate the probability of the established MOI phenotype, a discriminatory nomogram based on this model was developed (Figure 3). Examining the nomogram, it is seen that the highest discriminatory score determining the risk of MOI phenotype comes directly from the SIRI axis. The clinical reliability and discriminatory performance of this developed nomogram were evaluated using the Hosmer–Lemeshow Calibration Curve. It was observed that the actual MOI phenotype disease rates and the probabilities predicted by the nomogram were consistent with the 45-degree ideal reference line. In order to prove that the model does not merely replicate the existing dataset, the 1000-resampling bootstrap internal validation resulted in an optimism value of only +0.0060, which is negligible, confirming the strong discriminatory ability of the model. Univariable ROC analysis demonstrated that SIRI alone yielded an AUC of 0.919 (95% CI: 0.880–0.956) (Table 2). In comparison, the multivariable Firth-penalized model incorporating SIRI, age, sex, and disease duration achieved an apparent AUC of 0.922 (95% CI: 0.881–0.963) and an internal bootstrap optimism-corrected AUC of 0.912 (Table 3, Figure 3).

To increase the accessibility and usability of SIRI-based discriminatory nomograms in daily outpatient practice, a web-based, dynamic clinical decision support tool (Behçet Nomogram Calculator) has been designed. Clinicians can instantly calculate the individualized probability of MOI phenotype by entering the patient’s routine complete blood count data into the system via this open-access platform (https://behcetrisc.streamlit.app/, accessed on 6 September 2026). This interactive tool has enabled the transformation of the mathematical model obtained in our study into a practical screening tool.

## 4. Discussion

This study generally analyzes the role of SIRI, SII, AISI, NLR, PLR, and NMLR in identifying the MOI phenotype of Behçet’s disease (BD) from mucocutaneous BD. The most important finding of our study, which will contribute to the literature, is that the levels of Erythrocyte Sedimentation Rate (ESR) and C-Reactive Protein (CRP), commonly used in clinical practice, are statistically insufficient in differentiating mucocutaneous Behçet’s disease patients from the MOI phenotype (*p* > 0.05). In contrast, AISI, SIRI, and SII values were found to be significantly higher in the MOI phenotype BD group compared to the mucocutaneous BD group (*p* < 0.001). These results suggest that conventional laboratory parameters may be insufficient in reflecting the systemic inflammatory burden associated with established organ involvement in the course of BD, whereas novel biomarkers integrating the thrombo-inflammatory cascade at the cellular level may be much more sensitive guides in clinical follow-up.

In previous studies, these indices have been found to show strong correlations with disease activity in inflammatory pathologies such as rheumatoid arthritis, systemic lupus erythematosus, and ankylosing spondylitis [19,20,21]. In a recent study in BD, it was reported that the biomarkers SII, SIRI, and NPAR exhibited strong discrimination (AUC) of 0.87, 0.87, and 0.88 in differentiating major organ and especially vascular involvement from isolated mucocutaneous forms in BD patients [15]. These data clinically support the baseline hypothesis and findings of our study. However, the fact that the CRP (AUC: 0.563) and ESR (AUC: 0.552) curves remained within the 50% range indicates that in clinical practice, major organ involvement may be missed by monitoring only sedimentation rate and CRP.

The observed lack of significant differences in traditional acute phase reactants (CRP and ESR) between the study groups highlights the limited capacity of these conventional markers to reflect end-organ damage in Behçet’s disease. This finding may be largely attributable to the intensive use of conventional DMARDs and biological agents in the MOI group, which effectively suppresses systemic acute-phase responses. Remarkably, despite concurrent immunosuppressive therapies, SIRI remained significantly elevated and demonstrated high discriminatory performance. This suggests that SIRI may better capture systemic immune alterations that escape classical biomarker surveillance under treatment. This phenomenon parallels recent molecular observations in the literature; for example, Ecesoy et al. reported that SIRT1 levels did not significantly differ based on overall clinical activity scores (BDCAF) but were uniquely altered in patients with severe major organ involvement [22]. Consequently, our SIRI-based discriminatory nomogram offers a sensitive clinical decision-support tool for identifying vascular and organ-specific inflammatory burdens that are frequently overlooked by standard parameters.

Ocular involvement in BD is a common condition that can be the first sign of the disease and can lead to blindness. In a study, SII and SIRI values were found to be much higher in the group with uveitis due to Behçet’s disease than in the BD group without uveitis and the healthy control group [23]. The analyses revealed that a high SII level is independently associated with the presence of BD uveitis. With an SII AUC of 0.752, they reported a numerically higher discriminative value than traditional NLR (AUC = 0.690). In our study, the MOI phenotype group also included uveitis patients, and the SII value AUC = 0.883 was observed, which is consistent with the literature.

Recent studies have focused on the role of the SII, which integrates neutrophil, lymphocyte, and platelet counts to reflect systemic inflammation and thrombotic predisposition and the NLR in BD. In the review presented by Daood et al., it was emphasized that NLR showed a consistent correlation with disease activity. Furthermore, NLR values were found to show a consistent correlation with specific clinical signs, particularly mucocutaneous, uveitis, vascular, and joint involvement. Despite this, a standard cut-off value for NLR could not be found because it is not disease-specific and can be affected by factors such as age, gender, smoking or drug use [24]. In our study, optimized clear threshold values with a sensitivity of 84.27% using the Youden index were determined for use in clinical practice, with an NLR cut-off value ≥2.16. This contributes to addressing the lack of standard cut-offs in the literature by providing a clear threshold value based on major organ involvement.

In a study on the use of SII in determining disease activity, higher numbers of white blood cells, platelets and neutrophils; wider red blood cell distribution width; higher ESR, CRP and ferritin levels; and higher SII were observed in the active disease group [25]. In this study, the cut-off value for SII in determining BD activity was reported as 552 × 10^3^/mm^3^ (Sensitivity: 81%, Specificity: 82%, AUC: 0.84), and it was emphasized that levels above this value may be an indicator of severe inflammation. Our study supports the findings of Tanacan et al., but it was observed that the MOI phenotype exhibits higher inflammatory parameters. In our study, the SII cut-off value we determined for the MOI phenotype ofBehçet’s disease (≥653.59, AUC: 0.883) was observed to be significantly higher than the threshold value reported by Tanacan et al. (552) for overall disease activity. We hypothesize that the distinct thrombo-inflammatory pathophysiology of the MOI phenotype, particularly vascular and severe ocular attacks, inherently triggers a more profound systemic cellular immune activation compared to isolated mucocutaneous flares. This situation increases neutrophil and platelet multiplication in the formula, resulting in a higher inflammatory threshold proportional to disease severity and organ damage.

In our study, SIRI yielded the highest numerical point estimate for AUC (0.919, 95% CI: 0.880–0.956); however, formal paired ROC comparisons demonstrated that its discriminative performance was statistically comparable to that of AISI (AUC: 0.916; *p* = 0.800) and SII (AUC: 0.889; *p* = 0.211). A recent study in the literature compared HLA-B51-positive BD patients with healthy controls; NLR and SII values were found to be significant in the diagnosis of BD, while SIRI levels were not different from healthy controls (*p* = 0.894) [18]. The findings of our study explain this difference. In the study by Aci et al., the vast majority of patients were in the inactive disease phase (mean BDCAF score: 1.16), and none of the patients had neurological or intestinal involvement. Our study demonstrates that SIRI is not merely a diagnostic marker indicating the presence of disease, but rather a specific parameter strongly associated with the established MOI phenotype in a cross-sectional setting. The integration of monocyte counts into composite formulas, as seen in SIRI, provides a significant discriminative gain over traditional dual ratios such as NLR (*p* = 0.010), highlighting the contribution of monocyte-mediated inflammation in systemic involvement. The role of systemic inflammatory indices (NLR and SII) in BD has been discussed in the literature in recent years. Kılıç et al. investigated the value of markers such as NLR and SII in discriminating major organ and vascular involvement in BD [17]. In this study, in order to increase diagnostic power, they derived new indices by multiplying the inflammatory indices (NLR and SII) with CRP, an acute-phase reactant synthesized in the liver (IBI-NLR, IBI-SII), but they could only reach a maximum AUC value of 0.624 even for vascular involvement. Furthermore, it was reported that SII was not statistically significant in discriminatory major organ involvement (*p* > 0.05). Our findings, however, are completely different from this study. Our study not only demonstrated high diagnostic accuracy for NLR (AUC: 0.860) and SII (AUC: 0.883), but also proved that cellular indices can be powerfully discriminatory in specific subphenotypes, even without the need for CRP integration. The low AUC values in Kılıç et al.’s cohort are likely due to heterogeneity in the definition of major organ involvement and the clinical stages and immunosuppressive treatments received by the patients at the time of analysis. While our study demonstrated excellent discriminative accuracy for SIRI (AUC: 0.919), these findings must be interpreted critically when compared to recent, larger Behçet’s disease cohorts that have frequently reported more modest diagnostic performances for CBC-derived inflammatory indices. It is crucial not to assume that the high discrimination observed in our present cohort reveals a universally more accurate ‘true’ effect. Rather, the robust performance in our study may be heavily influenced by specific cohort characteristics. Differences in maintenance treatment protocols, baseline disease activity states, specific phenotype composition, geographic and genetic background, selection bias inherent to a single-center design, and statistical chance must all be rigorously considered. Therefore, the effect size observed here may be highly context-dependent, underscoring the absolute necessity for external validation in larger, prospective multi-center cohorts before assuming universal clinical applicability. In the literature, different modalities are being investigated to discriminate the pathological processes of BD. In the study by Alibaz-Oner et al., it was shown that common femoral vein (CFV) wall thickness is significantly increased in patients with BD, independent of major organ involvement and disease activity, and is a powerful tool in the diagnosis of the disease (with a cut-off value of ≥0.5 mm) [3]. This study also highlighted that CFV thickening, a structural finding, showed no correlation with classical acute phase reactants (ESR and CRP). Our findings confirm Alibaz-Oner et al.’s finding that classical inflammation markers (ESR, CRP) are insufficient to reflect BD pathology at the systemic cellular level. While Alibaz-Oner et al.’s data indicate that vascular wall thickening is an intrinsic and structural diagnostic feature of BD independent of organ involvement, our results demonstrate that cell-based indices such as SIRI and SII are dynamic markers that show a strong cross-sectional association with the established MOI phenotype in the course of the disease with high accuracy (AUC for SIRI: 0.919). Both studies, when examined together, conclude that ultrasonographic CFV thickness measurement is a complementary clinical tool in confirming the baseline diagnosis of BD, while next-generation monocyte-based indices such as SIRI are complementary tools in assessing the likelihood of concurrent major organ damage.

The literature indicates that routine comprehensive screening for all asymptomatic patients is not currently a feasible or recommended method [11]. However, in rheumatology practice, the important goal is to effectively monitor stable outpatients to distinguish those who might have underlying established major organ involvement. Cut-off values for SIRI (≥1.30), AISI (≥322), and SII (≥653.59), which are routine, readily calculated in seconds, and available in every healthcare facility like a complete blood count test, can provide clinicians with an accessible adjunctive tool for the cross-sectional discrimination of the MOI phenotype in daily practice and for planning effective therapeutic evaluations. One of the strongest aspects that distinguishes our study from similar studies in the literature is that it has been transformed into a free and dynamic online tool (https://behcetrisc.streamlit.app/, accessed on 6 September 2026) that eliminates the burden of manual calculations for physicians.

It is important to emphasize that SIRI is a mathematical combination of peripheral cell counts (neutrophilia, monocytosis, and lymphopenia); it is not a direct measure of endothelial dysfunction, thrombosis, neutrophil extracellular trap (NET) formation, or tissue ischemia. Because our study did not directly measure cytokines, endothelial activation markers, or thrombin generation, any mechanistic claims linking these composite indices directly to active vascular inflammation remain purely speculative.

## 5. Limitations

This study has some limitations. The cross-sectional design of our study and its reliance on a single-center patient population (Karaman Training and Research Hospital) are noted as factors limiting generalizability. Additionally, the lack of direct molecular measurements of cytokines, NETs, or endothelial activation markers limits our ability to draw definitive mechanistic conclusions regarding thrombo-inflammatory activity. Another limitation is that the asymptomatic patients in the mucocutaneous group did not undergo universal, standardized ophthalmological, vascular, neurological, or gastrointestinal screening evaluations, as this is not part of routine clinical care. Consequently, some patients classified as purely mucocutaneous might harbor unrecognized, asymptomatic organ involvement. This bias limits any definitive claims regarding the capacity of our CBC-derived indices and nomogram to detect truly subclinical disease. Furthermore, the lack of cumulative data on patients’ smoking status and subclinical external factors (mild viral infections, etc.) that may temporarily affect hemogram parameters in medical records is among the limitations of our study. In this study, due to the absence of a simultaneous and validated measure of disease activity (such as BDCAF) at the time of blood sampling, it is not possible to definitively distinguish the established MOI phenotype-specific inflammatory signature from potential residual or low-grade concurrent disease activity. Furthermore, current treatment protocols present a mathematically unresolvable methodological challenge in this cross-sectional design. Therapies differ significantly between the phenotypes; they strictly follow the development of MOI and directly influence leukocyte counts. As previously noted, adjusting for these treatments would introduce critical post-outcome or collider bias. Consequently, the CBC-derived indices evaluated in this cohort should be interpreted not as causal measures of natural, untreated disease activity but rather as real-world reflections of the patients’ net systemic state under varying standard-of-care pharmacotherapies. Although the proposed nomogram demonstrated excellent internal validation using bootstrap resampling, external validation in independent, prospective multi-center cohorts is essential before recommending this model for widespread routine clinical use. To fully validate the treatment response and dynamic follow-up value of these novel cellular biomarkers in BD management, large-scale and prospective multi-center cohort studies are needed. Finally, while bootstrap internal validation confirmed model stability, the proposed cut-offs must be regarded as exploratory until validated in independent, prospective longitudinal cohorts.

## 6. Conclusions

In conclusion, conventional acute-phase reactants (ESR, CRP) fail to reflect established major organ involvement in Behçet’s disease patients receiving maintenance therapies outside acute flares. In contrast, composite CBC-derived inflammatory indices, particularly SIRI, offer robust cross-sectional discriminatory performance. The developed SIRI-based nomogram provides a practical clinical stratification tool, although external prospective validation is required prior to routine clinical integration. Importantly, given the cross-sectional design of this study, the present data do not establish the prediction of future MOI, prognosis, subclinical vasculitis, or the benefit of biomarker-guided treatments.

## Figures and Tables

**Table 1:** Demographic, Clinical, and Laboratory Characteristics Across Discrete Clinical Phenotypes in Behçet’s Disease.

Variables	Mucocutaneous Group (n = 90)	Isolated Uveitis Group (n = 44)	Isolated Vascular Involvement Group (n = 27)	Uveitis + Vascular Involvement Group (n = 17)	*p*
Demographic and Clinical Characteristics					
Age (years)	42.50 [32.25–52.00]	45.00 [33.75–52.00]	45.00 [39.50–53.00]	42.00 [38.00–50.00]	0.529
Disease duration (years)	12.00 [7.00–20.00]	15.50 [8.00–22.50]	14.00 [12.00–25.00]	16.00 [12.00–22.00]	0.093
Male sex, n (%)	25 (27.8%)	26 (59.1%)	17 (63.0%)	14 (82.4%)	<0.001
Female sex, n (%)	65 (72.2%)	18 (40.9%)	10 (37.0%)	3 (17.6%)	<0.001
Medical Treatments, n (%)					
Colchicine	87 (96.7%)	34 (77.3%)	21 (77.8%)	7 (41.2%)	<0.001
Conventional DMARDs	23 (25.6%)	24 (54.5%)	19 (70.4%)	11 (64.7%)	<0.001
Biologic agents (anti-TNF)	0 (0.0%)	13 (29.5%)	5 (18.5%)	8 (47.1%)	<0.001
Acute Phase Reactants					
ESR (mm/h)	7.00 [4.00–11.00]	9.00 [4.00–13.50]	11.00 [4.00–18.00]	6.00 [3.00–18.00]	0.388
CRP (mg/L)	2.35 [1.52–5.38]	4.35 [1.27–7.30]	4.40 [1.10–7.05]	2.90 [1.70–11.10]	0.533
Complete Blood Count Parameters					
Neutrophils (10^3^/μL)	4.52 [3.41–5.18]	5.84 [5.07–7.01]	5.97 [4.79–6.89]	6.88 [5.78–7.64]	<0.001
Platelets (10^3^/μL)	259.50 [228.50–298.75]	290.50 [252.25–340.50]	339.00 [252.50–445.00]	330.00 [265.00–378.00]	<0.001
Lymphocytes (10^3^/μL)	2.33 [1.96–2.90]	2.10 [1.81–2.81]	2.17 [1.42–2.67]	2.33 [1.98–2.80]	0.168
Monocytes (10^3^/μL)	0.45 [0.39–0.53]	0.62 [0.50–0.69]	0.51 [0.47–0.60]	0.57 [0.41–0.71]	<0.001
Composite CBC-Derived Inflammatory Indices					
SIRI	0.83 [0.65–1.01]	1.68 [1.33–1.92]	1.49 [1.24–1.88]	1.75 [1.30–2.12]	<0.001
SII	465.09 [362.43–617.75]	808.45 [676.41–999.22]	1046.87 [745.75–1239.49]	983.02 [800.8–1097.58]	<0.001
AISI	217.71 [151.34–282.23]	493.51 [395.05–590.24]	561.95 [346.73–679.03]	576.64 [403.04–750.82]	<0.001
NLR	1.87 [1.51–2.23]	2.82 [2.47–3.05]	2.86 [2.44–3.48]	2.58 [2.31–3.15]	<0.001

Abbreviations: DMARDs, Disease-Modifying Antirheumatic Drugs; ESR, Erythrocyte Sedimentation Rate; CRP, C-Reactive Protein; SIRI, Systemic Inflammation Response Index; SII, Systemic Immuno-Inflammation Index; AISI, Aggregate Index of Systemic Inflammation; NLR, Neutrophil-to-Lymphocyte Ratio.

**Table 2:** Diagnostic Accuracy and Phenotype-Specific ROC Analysis of Inflammatory Parameters in Discriminating Major Organ Involvement from Pure Mucocutaneous Behçet’s Disease.

Parameter	MOI BD vs. Mucocutaneous			Isolated Uveitis vs. Mucocutaneous	Isolated Vasculitis vs. Mucocutaneous	Uveitis + Vasculitis vs. Mucocutaneous
	AUC (95% CI)	Cut-Off	Sens./Spec. (%)	AUC (95% CI)	AUC (95% CI)	AUC (95% CI)
SIRI	0.919 (0.880–0.956)	≥1.30	75.0%/93.3%	0.935 (0.890–0.969)	0.924 (0.864–0.965)	0.871 (0.727–0.982)
AISI	0.916 (0.874–0.955)	≥322.51	85.2%/85.6%	0.918 (0.860–0.963)	0.944 (0.899–0.977)	0.866 (0.719–0.988)
SII	0.889 (0.843–0.933)	≥653.59	84.1%/82.2%	0.863 (0.783–0.923)	0.944 (0.891–0.983)	0.871 (0.725–0.996)
NMLR	0.873 (0.826–0.923)	≥2.35	89.8%/72.2%	0.881 (0.810–0.936)	0.893 (0.820–0.946)	0.823 (0.694–0.924)
NLR	0.865 (0.811–0.916)	≥2.16	85.2%/73.3%	0.871 (0.796–0.929)	0.888 (0.811–0.942)	0.813 (0.687–0.915)
PLR	0.736 (0.659–0.804)	≥118.67	79.5%/62.2%	0.665 (0.568–0.756)	0.850 (0.759–0.924)	0.740 (0.584–0.871)
CRP	0.562 (0.483–0.646)	≥5.50	42.0%/76.7%	0.565 (0.450–0.669)	0.550 (0.414–0.689)	0.574 (0.422–0.738)
ESR	0.558 (0.478–0.640)	≥12.00	42.0%/75.6%	0.553 (0.447–0.660)	0.604 (0.484–0.735)	0.499 (0.319–0.693)

Abbreviations: AISI, Aggregate Index of Systemic Inflammation; AUC, Area Under the Curve; CI, Confidence Interval; CRP, C-Reactive Protein; ESR, Erythrocyte Sedimentation Rate; MOI, Major Organ Involvement; NLR, Neutrophil-to-Lymphocyte Ratio; NMLR, Neutrophil-Monocyte-Lymphocyte Ratio; PLR, Platelet-to-Lymphocyte Ratio; Sens, Sensitivity; SII, Systemic Immuno-Inflammation Index; SIRI, Systemic Inflammatory Response Index; Spec, Specificity.

**Table 3:** Discriminative Performance of SIRI Across Demographic and Clinical Subgroups in Identifying Established Major Organ Involvement in Behçet’s Disease.

Clinical Subgroups	Total Patients (n)	Patients with Established MOI, n (%)	SIRI AUC (95% CI)
Overall Cohort	178	88 (49.4%)	0.919 (0.879–0.955)
Age Category			
≤40 years	70	27 (38.6%)	0.929 (0.867–0.978)
>40 years	108	61 (56.5%)	0.920 (0.865–0.963)
Sex			
Male	82	57 (69.5%)	0.881 (0.802–0.946)
Female	96	31 (32.3%)	0.944 (0.898–0.979)
Disease Duration			
≤5 years	27	7 (25.9%)	0.936 (0.821–1.000)
>5 years	151	81 (53.6%)	0.915 (0.869–0.957)

Abbreviations: AUC, Area Under the Curve; CI, Confidence Interval; MOI, Major Organ Involvement; SIRI, Systemic Inflammatory Response Index.

**Table 4:** Multivariable Firth’s Penalized Logistic Regression Model for Discriminating Established Major Organ Involvement in Behçet’s Disease.

Predictor Variables	β Coefficient	Standard Error (SE)	Adjusted Odds Ratio (aOR)	95% Confidence Interval	*p*
Intercept	−5.9981	1.3486	—	—	<0.001
SIRI	4.5019	0.7153	90.19	22.20–366.44	<0.001
Age (per year)	0.0058	0.0336	1.01	0.94–1.07	0.862
Sex (Male vs. Female)	0.5519	0.4532	1.74	0.71–4.22	0.223
Disease Duration (per year)	0.0086	0.0396	1.01	0.93–1.09	0.827

Model Performance and Internal Validation. Apparent Discrimination (C-index/AUC): 0.922 (95% CI: 0.881–0.963). 1000-Sample Bootstrap Optimism: +0.0098; Optimism-Corrected AUC: 0.912. Brier Calibration Score: 0.115. Abbreviations: aOR, Adjusted Odds Ratio; AUC, Area Under the Curve; CI, Confidence Interval; MOI, Major Organ Involvement; SE, Standard Error; SIRI, Systemic Inflammatory Response Index.

## Data Availability

The datasets generated and analyzed during the current study are not publicly available due to patient privacy considerations but are available from the corresponding author upon reasonable request.

## Abbreviations

AISI	Systemic Inflammation Aggregate Index
AUC	Area Under the Curve
BD	Behçet’s Disease
BDCAF	Behçet’s Disease Current Activity Form
CFV	Common Femoral Vein
CRP	C-Reactive Protein
DMARDs	Disease-Modifying Antirheumatic Drugs
ESR	Erythrocyte Sedimentation Rate
ICBD	International Criteria for Behçet’s Disease
IQR	Interquartile Range
MOI	Major Organ Involvement
NLR	Neutrophil-to-Lymphocyte Ratio
NMLR	Neutrophil–Monocyte–Lymphocyte Ratio
OR	Odds Ratio
PLR	Platelet-to-Lymphocyte Ratio
ROC	Receiver Operating Characteristic
SII	Systemic Immuno-Inflammation Index
SIRI	Systemic Inflammatory Response Index
